# Supplementary material for: Continuous Subcutaneous Insulin Infusion (CSII) Combined with Oral Glucose-Lowering Drugs in Type 2 Diabetes: A Systematic Review and Network Meta-Analysis of Randomized, Controlled Trials
Source: Pharmaceuticals (Basel). 2022 Jul 30;15(8):953. doi: 10.3390/ph15080953 (PMC9412496; doi:10.3390/ph15080953)
Supplement: Supplementary file 1 [file pharmaceuticals-15-00953-s001.zip › pharmaceuticals-1786209-supplementary.pdf]

Supplementary data

File S1: Search strategy for RCTs assessing the effects of insulin infusion combined with drugs in the treatment of patients with type 2 diabete

Cochrane:

ID Search

#1 (Diabetes Mellitus, Insulin-Dependent):ab,ti,kw OR (Diabetes Mellitus, Ketosis-Resistant):ab,ti,kw OR (Diabetes Mellitus, Ketosis Resistant):ab,ti,kw OR (Ketosis-Resistant Diabetes Mellitus):ab,ti,kw OR (Diabetes Mellitus, Non Insulin Dependent):ab,ti,kw OR (Diabetes Mellitus, Non-Insulin-Dependent):ab,ti,kw OR (Non-Insulin-Dependent Diabetes Mellitus):ab,ti,kw OR (Diabetes Mellitus, Stable):ab,ti,kw OR (Stable Diabetes Mellitus):ab,ti,kw OR (JDiabetes Mellitus, Type II):ab,ti OR (NIDDM):ab,ti,kw OR (Diabetes Mellitus, Noninsulin Dependent):ab,ti,kw OR (Diabetes Mellitus, Maturity-Onset):ab,ti,kw OR (Diabetes Mellitus, Maturity Onset):ab,ti,kw OR (Maturity-Onset Diabetes Mellitus):ab,ti,kw OR (Maturity Onset Diabetes Mellitus):ab,ti,kw OR (MODY):ab,ti,kw OR (Diabetes Mellitus, Slow-Onset):ab,ti,kw OR (Diabetes Mellitus, Slow Onset):ab,ti,kw OR (Slow-Onset Diabetes Mellitus):ab,ti,kw OR (Type 2 Diabetes Mellitus):ab,ti,kw OR (Noninsulin-Dependent Diabetes Mellitus):ab,ti,kw OR (Noninsulin Dependent Diabetes Mellitus):ab,ti,kw OR (Maturity-Onset Diabetes):ab,ti,kw OR (Diabetes, Maturity-Onset):ab,ti,kw OR (Maturity Onset Diabetes):ab,ti,kw OR (Type 2 Diabetes):ab,ti,kw OR (Diabetes, Type 2):ab,ti,kw OR (Diabetes Mellitus, Adult-Onset):ab,ti,kw OR (Adult-Onset Diabetes Mellitus):ab,ti,kw OR (Diabetes Mellitus, Adult Onset):ab,ti,kw

#2 (Infusion System, Insulin):ab,ti,kw OR (Infusion Systems, Insulin):ab,ti,kw OR (Insulin Infusion System):ab,ti,kw OR (System, Insulin Infusion):ab,ti,kw OR (Systems, Insulin Infusion):ab,ti,kw OR (Implantable Programmable Insulin Pump):ab,ti,kw OR (Programmable Implantable Insulin Pump):ab,ti,kw OR (Pump, Programmable Implantable Insulin):ab,ti,kw OR (Insulin Pump, Programmable Implantable):ab,ti,kw OR (Pancreas, Artificial Endocrine):ab,ti,kw OR (Artificial Endocrine Pancreas):ab,ti,kw OR (Endocrine Pancreas, Artificial):ab,ti,kw OR (beta Cell, Artificial):ab,ti,kw OR (Artificial beta Cell):ab,ti,kw OR (Artificial beta Cells):ab,ti,kw OR (Cell, Artificial beta):ab,ti,kw OR (Cells, Artificial beta):ab,ti,kw OR (beta Cells, Artificial):ab,ti,kw

#3 (Dimethylbiguanidine):ab,ti,kw OR (Dimethylguanylguanidine):ab,ti,kw OR (Glucophage): ab, ti,kw OR (Metformin Hydrochloride):ab, ti,kw OR (Hydrochloride, Metformin): ab, ti,kw OR (Metformin HCl):ab,ti,kw OR (HCl, Metformin):ab,ti,kw

#4 (Glumida):ab,ti,kw OR (Glucobay):ab,ti,kw OR (Glucor):ab,ti,kw OR (Bay g 5421):ab,ti,kw OR (Prandase):ab,ti,kw OR (Precose):ab,ti,kw

#5 (Sodium Glucose Transporter 2 Inhibitors):ab,ti,kw OR (Sodium-Glucose Transporter 2 Inhibitor):ab,ti,kw OR (Sodium Glucose Transporter 2 Inhibitor):ab,ti,kw OR (SGLT-2 Inhibitors):ab,ti,kw OR (SGLT 2 Inhibitors):ab,ti,kw OR (Gliflozins):ab,ti,kw OR (SGLT2 Inhibitors):ab,ti,kw OR (Gliflozin):ab,ti,kw OR (SGLT-2 Inhibitor):ab,ti,kw OR (Inhibitor, SGLT-2):ab,ti,kw OR (SGLT 2 Inhibitor):ab,ti,kw OR (SGLT2 Inhibitor):ab,ti,kw OR (Inhibitor, SGLT2):ab,ti,kw OR (Canagliflozin):ab,ti,kw

#6 (Glucagon Like Peptide 1 Receptor):ab,ti,kw OR (Peptide-1 Receptor, Glucagon-Like):ab,ti,kw OR (Receptor, Glucagon-Like Peptide-1):ab,ti,kw OR (GLP-1R Receptor):ab,ti,kw OR (GLP 1R Receptor):ab,ti,kw OR (Receptor, GLP-1R):ab,ti,kw OR (GLP1R Protein):ab,ti,kw OR (Protein, GLP1R):ab,ti,kw OR (GLP-1 Receptor):ab,ti,kw OR (GLP 1 Receptor):ab,ti,kw OR (Receptor, GLP-1):ab,ti,kw OR (GLP1R Receptor):ab,ti,kw OR (Receptor, GLP1R):ab,ti,kw

#7 #2 or #3 or #4 or #5 or #6

#8 (randomized controlled trial):ab,ti,kw OR (randomized):ab,ti,kw OR (placebo):ab,ti OR (RCT):ab,ti,kw

#9 #1 or #7 or #8

PubMed

(1) CSII combined with TZDs

((("Diabetes Mellitus, Type 2"[Mesh]) OR (((((((((((((((((((((((((((((((Diabetes Mellitus, Noninsulin-Dependent[Title/Abstract]) OR (Diabetes Mellitus, Ketosis-Resistant[Title/Abstract])) OR (Diabetes Mellitus, Ketosis Resistant[Title/Abstract])) OR (Ketosis-Resistant Diabetes Mellitus[Title/Abstract])) OR (Diabetes Mellitus, Non Insulin Dependent[Title/Abstract])) OR (Diabetes Mellitus, Non-Insulin-Dependent[Title/Abstract])) OR (Non-Insulin-Dependent Diabetes Mellitus[Title/Abstract])) OR (Diabetes Mellitus, Stable[Title/Abstract])) OR (Stable Diabetes Mellitus[Title/Abstract])) OR (Diabetes Mellitus, Type II[Title/Abstract])) OR (NIDDM[Title/Abstract])) OR (Diabetes Mellitus, Noninsulin Dependent[Title/Abstract])) OR (Diabetes Mellitus, Maturity-Onset[Title/Abstract])) OR (Diabetes Mellitus, Maturity Onset[Title/Abstract])) OR (Maturity-Onset Diabetes Mellitus[Title/Abstract])) OR (Maturity Onset Diabetes Mellitus[Title/Abstract])) OR (MODY[Title/Abstract])) OR (Diabetes Mellitus, Slow-Onset[Title/Abstract])) OR (Diabetes Mellitus, Slow Onset[Title/Abstract])) OR (Slow-Onset Diabetes Mellitus[Title/Abstract])) OR

(Type 2 Diabetes Mellitus[Title/Abstract])) OR (Noninsulin-Dependent Diabetes Mellitus[Title/Abstract])) OR (Noninsulin Dependent Diabetes Mellitus[Title/Abstract])) OR (Maturity-Onset Diabetes[Title/Abstract])) OR (Diabetes, Maturity-Onset[Title/Abstract])) OR (Maturity Onset Diabetes[Title/Abstract])) OR (Type 2 Diabetes[Title/Abstract])) OR (Diabetes, Type 2[Title/Abstract])) OR (Diabetes Mellitus, Adult-Onset[Title/Abstract])) OR (Adult-Onset Diabetes Mellitus[Title/Abstract])) OR (Diabetes Mellitus, Adult Onset[Title/Abstract]))) AND ((("Insulin Infusion Systems"[Mesh]) OR ((((((((((((((((((Infusion System, Insulin[Title/Abstract]) OR (Infusion Systems, Insulin[Title/Abstract])) OR (Insulin Infusion System[Title/Abstract])) OR (System, Insulin Infusion[Title/Abstract])) OR (Systems, Insulin Infusion[Title/Abstract])) OR (Implantable Programmable Insulin Pump[Title/Abstract])) OR (Programmable Implantable Insulin Pump[Title/Abstract])) OR (Pump, Programmable Implantable Insulin[Title/Abstract])) OR (Insulin Pump, Programmable Implantable[Title/Abstract])) OR (Pancreas, Artificial Endocrine[Title/Abstract])) OR (Artificial Endocrine Pancreas[Title/Abstract])) OR (Endocrine Pancreas, Artificial[Title/Abstract])) OR (beta Cell, Artificial[Title/Abstract])) OR (Artificial beta Cell[Title/Abstract])) OR (Artificial beta Cells[Title/Abstract])) OR (Cell, Artificial beta[Title/Abstract])) OR (Cells, Artificial beta[Title/Abstract])) OR (beta Cells, Artificial[Title/Abstract]))) AND (("Thiazolidinediones"[Mesh]) OR (Glitazones[Title/Abstract])))) AND (randomized controlled trial [Publication Type] OR randomized [Title/Abstract] OR placebo [Title/Abstract])

(2) CSII combined with metformin

(((("Diabetes Mellitus, Type 2"[Mesh]) OR (((((((((((((((((((((((((((((((Diabetes Mellitus, Noninsulin-Dependent[Title/Abstract]) OR (Diabetes Mellitus, Ketosis-Resistant[Title/Abstract])) OR (Diabetes Mellitus, Ketosis Resistant[Title/Abstract])) OR (Ketosis-Resistant Diabetes Mellitus[Title/Abstract])) OR (Diabetes Mellitus, Non Insulin Dependent[Title/Abstract])) OR (Diabetes Mellitus, Non-Insulin-Dependent[Title/Abstract])) OR (Non-Insulin-Dependent Diabetes Mellitus[Title/Abstract])) OR (Diabetes Mellitus, Stable[Title/Abstract])) OR (Stable Diabetes Mellitus[Title/Abstract])) OR (Diabetes Mellitus, Type II[Title/Abstract])) OR (NIDDM[Title/Abstract])) OR (Diabetes Mellitus, Noninsulin Dependent[Title/Abstract])) OR (Diabetes Mellitus, Maturity-Onset[Title/Abstract])) OR (Diabetes Mellitus, Maturity Onset[Title/Abstract])) OR (Maturity-Onset Diabetes Mellitus[Title/Abstract])) OR (Maturity Onset Diabetes Mellitus[Title/Abstract])) OR (MODY[Title/Abstract])) OR (Diabetes Mellitus, Slow-Onset[Title/Abstract])) OR (Diabetes Mellitus, Slow Onset[Title/Abstract])) OR (Slow-Onset Diabetes Mellitus[Title/Abstract])) OR (Type 2 Diabetes Mellitus[Title/Abstract])) OR (Noninsulin-Dependent Diabetes Mellitus[Title/Abstract])) OR (Noninsulin Dependent Diabetes Mellitus[Title/Abstract])) OR (Maturity-Onset Diabetes[Title/Abstract])) OR (Diabetes, Maturity-Onset[Title/Abstract])) OR (Maturity Onset Diabetes[Title/Abstract])) OR (Type 2 Diabetes[Title/Abstract])) OR (Diabetes, Type 2[Title/Abstract])) OR (Diabetes Mellitus, Adult-Onset[Title/Abstract])) OR (Adult-

Onset Diabetes Mellitus[Title/Abstract])) OR (Diabetes Mellitus, Adult Onset[Title/Abstract]))) AND (("Insulin Infusion Systems"[Mesh]) OR (((((((((((((((((((Infusion System, Insulin[Title/Abstract]) OR (Infusion Systems, Insulin[Title/Abstract])) OR (Insulin Infusion System[Title/Abstract])) OR (System, Insulin Infusion[Title/Abstract])) OR (Systems, Insulin Infusion[Title/Abstract])) OR (Implantable Programmable Insulin Pump[Title/Abstract])) OR (Programmable Implantable Insulin Pump[Title/Abstract])) OR (Pump, Programmable Implantable Insulin[Title/Abstract])) OR (Insulin Pump, Programmable Implantable[Title/Abstract])) OR (Pancreas, Artificial Endocrine[Title/Abstract])) OR (Artificial Endocrine Pancreas[Title/Abstract])) OR (Endocrine Pancreas, Artificial[Title/Abstract])) OR (beta Cell, Artificial[Title/Abstract])) OR (Artificial beta Cell[Title/Abstract])) OR (Artificial beta Cells[Title/Abstract])) OR (Cell, Artificial beta[Title/Abstract])) OR (Cells, Artificial beta[Title/Abstract])) OR (beta Cells, Artificial[Title/Abstract])))) AND (("Metformin"[Mesh]) OR (((((((Dimethylbiguanidine[Title/Abstract]) OR (Dimethylguanylguanidine[Title/Abstract])) OR (Glucophage[Title/Abstract])) OR (Metformin Hydrochloride[Title/Abstract])) OR (Hydrochloride, Metformin[Title/Abstract])) OR (Metformin HCl[Title/Abstract])) OR (HCl, Metformin[Title/Abstract])))) AND (randomized controlled trial [Publication Type] OR randomized [Title/Abstract] OR placebo [Title/Abstract])

(3) CSII combined with acarbose

(((("Diabetes Mellitus, Type 2"[Mesh]) OR ((((((((((((((((((((((((((((((((Diabetes Mellitus, Noninsulin-Dependent[Title/Abstract]) OR (Diabetes Mellitus, Ketosis-Resistant[Title/Abstract])) OR (Diabetes Mellitus, Ketosis Resistant[Title/Abstract])) OR (Ketosis-Resistant Diabetes Mellitus[Title/Abstract])) OR (Diabetes Mellitus, Non Insulin Dependent[Title/Abstract])) OR (Diabetes Mellitus, Non-Insulin-Dependent[Title/Abstract])) OR (Non-Insulin-Dependent Diabetes Mellitus[Title/Abstract])) OR (Diabetes Mellitus, Stable[Title/Abstract])) OR (Stable Diabetes Mellitus[Title/Abstract])) OR (Diabetes Mellitus, Type II[Title/Abstract])) OR (NIDDM[Title/Abstract])) OR (Diabetes Mellitus, Noninsulin Dependent[Title/Abstract])) OR (Diabetes Mellitus, Maturity-Onset[Title/Abstract])) OR (Diabetes Mellitus, Maturity Onset[Title/Abstract])) OR (Maturity-Onset Diabetes Mellitus[Title/Abstract])) OR (Maturity Onset Diabetes Mellitus[Title/Abstract])) OR (MODY[Title/Abstract])) OR (Diabetes Mellitus, Slow-Onset[Title/Abstract])) OR (Diabetes Mellitus, Slow Onset[Title/Abstract])) OR (Slow-Onset Diabetes Mellitus[Title/Abstract])) OR (Type 2 Diabetes Mellitus[Title/Abstract])) OR (Noninsulin-Dependent Diabetes Mellitus[Title/Abstract])) OR (Noninsulin Dependent Diabetes Mellitus[Title/Abstract])) OR (Maturity-Onset Diabetes[Title/Abstract])) OR (Diabetes, Maturity-Onset[Title/Abstract])) OR (Maturity Onset Diabetes[Title/Abstract])) OR (Type 2 Diabetes[Title/Abstract])) OR (Diabetes, Type 2[Title/Abstract])) OR (Diabetes Mellitus, Adult-Onset[Title/Abstract])) OR (Adult-Onset Diabetes Mellitus[Title/Abstract])) OR (Diabetes Mellitus, Adult Onset[Title/Abstract]))) AND (("Insulin Infusion Systems"[Mesh]) OR (((((((((((((((((((Infusion System,

Insulin[Title/Abstract]) OR (Infusion Systems, Insulin[Title/Abstract])) OR (Insulin Infusion System[Title/Abstract])) OR (System, Insulin Infusion[Title/Abstract])) OR (Systems, Insulin Infusion[Title/Abstract])) OR (Implantable Programmable Insulin Pump[Title/Abstract])) OR (Programmable Implantable Insulin Pump[Title/Abstract])) OR (Pump, Programmable Implantable Insulin[Title/Abstract])) OR (Insulin Pump, Programmable Implantable[Title/Abstract])) OR (Pancreas, Artificial Endocrine[Title/Abstract])) OR (Artificial Endocrine Pancreas[Title/Abstract])) OR (Endocrine Pancreas, Artificial[Title/Abstract])) OR (beta Cell, Artificial[Title/Abstract])) OR (Artificial beta Cell[Title/Abstract])) OR (Artificial beta Cells[Title/Abstract])) OR (Cell, Artificial beta[Title/Abstract])) OR (Cells, Artificial beta[Title/Abstract])) OR (beta Cells, Artificial[Title/Abstract])))) AND (randomized controlled trial [Publication Type] OR randomized [Title/Abstract] OR placebo [Title/Abstract])) AND (("Acarbose"[Mesh]) OR ((((((Glumida[Title/Abstract]) OR (Glucobay[Title/Abstract])) OR (Glucor[Title/Abstract])) OR (Bay g 5421[Title/Abstract])) OR (Prandase[Title/Abstract])) OR (Precose[Title/Abstract])))

(4) CSII combined with GLP-1

(((("Diabetes Mellitus, Type 2"[Mesh]) OR (((((((((((((((((((((((((((((((Diabetes Mellitus, Noninsulin-Dependent[Title/Abstract]) OR (Diabetes Mellitus, Ketosis-Resistant[Title/Abstract])) OR (Diabetes Mellitus, Ketosis Resistant[Title/Abstract])) OR (Ketosis-Resistant Diabetes Mellitus[Title/Abstract])) OR (Diabetes Mellitus, Non Insulin Dependent[Title/Abstract])) OR (Diabetes Mellitus, Non-Insulin-Dependent[Title/Abstract])) OR (Non-Insulin-Dependent Diabetes Mellitus[Title/Abstract])) OR (Diabetes Mellitus, Stable[Title/Abstract])) OR (Stable Diabetes Mellitus[Title/Abstract])) OR (Diabetes Mellitus, Type II[Title/Abstract])) OR (NIDDM[Title/Abstract])) OR (Diabetes Mellitus, Noninsulin Dependent[Title/Abstract])) OR (Diabetes Mellitus, Maturity-Onset[Title/Abstract])) OR (Diabetes Mellitus, Maturity Onset[Title/Abstract])) OR (Maturity-Onset Diabetes Mellitus[Title/Abstract])) OR (Maturity Onset Diabetes Mellitus[Title/Abstract])) OR (MODY[Title/Abstract])) OR (Diabetes Mellitus, Slow-Onset[Title/Abstract])) OR (Diabetes Mellitus, Slow Onset[Title/Abstract])) OR (Slow-Onset Diabetes Mellitus[Title/Abstract])) OR (Type 2 Diabetes Mellitus[Title/Abstract])) OR (Noninsulin-Dependent Diabetes Mellitus[Title/Abstract])) OR (Noninsulin Dependent Diabetes Mellitus[Title/Abstract])) OR (Maturity-Onset Diabetes[Title/Abstract])) OR (Diabetes, Maturity-Onset[Title/Abstract])) OR (Maturity Onset Diabetes[Title/Abstract])) OR (Type 2 Diabetes[Title/Abstract])) OR (Diabetes, Type 2[Title/Abstract])) OR (Diabetes Mellitus, Adult-Onset[Title/Abstract])) OR (Adult-Onset Diabetes Mellitus[Title/Abstract])) OR (Diabetes Mellitus, Adult Onset[Title/Abstract]))) AND (("Insulin Infusion Systems"[Mesh]) OR ((((((((((((((((((Infusion System, Insulin[Title/Abstract]) OR (Infusion Systems, Insulin[Title/Abstract])) OR (Insulin Infusion System[Title/Abstract])) OR (System, Insulin Infusion[Title/Abstract])) OR (Systems, Insulin Infusion[Title/Abstract])) OR (Implantable Programmable Insulin Pump[Title/Abstract])) OR (Programmable Implantable Insulin Pump[Title/Abstract])) OR (Pump, Programmable

Implantable Insulin[Title/Abstract])) OR (Insulin Pump, Programmable Implantable[Title/Abstract])) OR (Pancreas, Artificial Endocrine[Title/Abstract])) OR (Artificial Endocrine Pancreas[Title/Abstract])) OR (Endocrine Pancreas, Artificial[Title/Abstract])) OR (beta Cell, Artificial[Title/Abstract])) OR (Artificial beta Cell[Title/Abstract])) OR (Artificial beta Cells[Title/Abstract])) OR (Cell, Artificial beta[Title/Abstract])) OR (Cells, Artificial beta[Title/Abstract])) OR (beta Cells, Artificial[Title/Abstract])))) AND (randomized controlled trial [Publication Type] OR randomized [Title/Abstract] OR placebo [Title/Abstract])) AND (("Glucagon-Like Peptide-1 Receptor"[Mesh]) OR ((((((((((((Glucagon Like Peptide 1 Receptor[Title/Abstract]) OR (Peptide-1 Receptor, Glucagon-Like[Title/Abstract])) OR (Receptor, Glucagon-Like Peptide-1[Title/Abstract])) OR (GLP-1R Receptor[Title/Abstract])) OR (GLP 1R Receptor[Title/Abstract])) OR (Receptor, GLP-1R[Title/Abstract])) OR (GLP1R Protein[Title/Abstract])) OR (Protein, GLP1R[Title/Abstract])) OR (GLP-1 Receptor[Title/Abstract])) OR (GLP 1 Receptor[Title/Abstract])) OR (Receptor, GLP-1[Title/Abstract])) OR (GLP1R Receptor[Title/Abstract])) OR (Receptor, GLP1R[Title/Abstract])))

(5) CSII combined with SGLT-2

(((("Diabetes Mellitus, Type 2"[Mesh]) OR ((((((((((((((((((((((((((((((Diabetes Mellitus, Noninsulin-Dependent[Title/Abstract]) OR (Diabetes Mellitus, Ketosis-Resistant[Title/Abstract])) OR (Diabetes Mellitus, Ketosis Resistant[Title/Abstract])) OR (Ketosis-Resistant Diabetes Mellitus[Title/Abstract])) OR (Diabetes Mellitus, Non Insulin Dependent[Title/Abstract])) OR (Diabetes Mellitus, Non-Insulin-Dependent[Title/Abstract])) OR (Non-Insulin-Dependent Diabetes Mellitus[Title/Abstract])) OR (Diabetes Mellitus, Stable[Title/Abstract])) OR (Stable Diabetes Mellitus[Title/Abstract])) OR (Diabetes Mellitus, Type II[Title/Abstract])) OR (NIDDM[Title/Abstract])) OR (Diabetes Mellitus, Noninsulin Dependent[Title/Abstract])) OR (Diabetes Mellitus, Maturity-Onset[Title/Abstract])) OR (Diabetes Mellitus, Maturity Onset[Title/Abstract])) OR (Maturity-Onset Diabetes Mellitus[Title/Abstract])) OR (Maturity Onset Diabetes Mellitus[Title/Abstract])) OR (MODY[Title/Abstract])) OR (Diabetes Mellitus, Slow-Onset[Title/Abstract])) OR (Diabetes Mellitus, Slow Onset[Title/Abstract])) OR (Slow-Onset Diabetes Mellitus[Title/Abstract])) OR (Type 2 Diabetes Mellitus[Title/Abstract])) OR (Noninsulin-Dependent Diabetes Mellitus[Title/Abstract])) OR (Noninsulin Dependent Diabetes Mellitus[Title/Abstract])) OR (Maturity-Onset Diabetes[Title/Abstract])) OR (Diabetes, Maturity-Onset[Title/Abstract])) OR (Maturity Onset Diabetes[Title/Abstract])) OR (Type 2 Diabetes[Title/Abstract])) OR (Diabetes, Type 2[Title/Abstract])) OR (Diabetes Mellitus, Adult-Onset[Title/Abstract])) OR (Adult-Onset Diabetes Mellitus[Title/Abstract])) OR (Diabetes Mellitus, Adult Onset[Title/Abstract]))) AND (("Insulin Infusion Systems"[Mesh]) OR (((((((((((((((((Infusion System, Insulin[Title/Abstract]) OR (Infusion Systems, Insulin[Title/Abstract])) OR (Insulin Infusion System[Title/Abstract])) OR (System, Insulin Infusion[Title/Abstract])) OR (Systems, Insulin

Infusion[Title/Abstract])) OR (Implantable Programmable Insulin Pump[Title/Abstract])) OR (Programmable Implantable Insulin Pump[Title/Abstract])) OR (Pump, Programmable Implantable Insulin[Title/Abstract])) OR (Insulin Pump, Programmable Implantable[Title/Abstract])) OR (Pancreas, Artificial Endocrine[Title/Abstract])) OR (Artificial Endocrine Pancreas[Title/Abstract])) OR (Endocrine Pancreas, Artificial[Title/Abstract])) OR (beta Cell, Artificial[Title/Abstract])) OR (Artificial beta Cell[Title/Abstract])) OR (Artificial beta Cells[Title/Abstract])) OR (Cell, Artificial beta[Title/Abstract])) OR (Cells, Artificial beta[Title/Abstract])) OR (beta Cells, Artificial[Title/Abstract])))) AND (randomized controlled trial [Publication Type] OR randomized [Title/Abstract] OR placebo [Title/Abstract])) AND (("Sodium-Glucose Transporter 2 Inhibitors"[Mesh]) OR (((((((((((((Sodium Glucose Transporter 2 Inhibitors[Title/Abstract]) OR (Sodium-Glucose Transporter 2 Inhibitor[Title/Abstract])) OR (Sodium Glucose Transporter 2 Inhibitor[Title/Abstract])) OR (SGLT-2 Inhibitors[Title/Abstract])) OR (SGLT 2 Inhibitors[Title/Abstract])) OR (Gliflozins[Title/Abstract])) OR (SGLT2 Inhibitors[Title/Abstract])) OR (Gliflozin[Title/Abstract])) OR (SGLT-2 Inhibitor[Title/Abstract])) OR (Inhibitor, SGLT-2[Title/Abstract])) OR (SGLT 2 Inhibitor[Title/Abstract])) OR (SGLT2 Inhibitor[Title/Abstract])) OR (Inhibitor, SGLT2[Title/Abstract])))

(6) CSII combined with DPP-4 inhibitor

((("Diabetes Mellitus, Type 2"[Mesh]) OR (((((((((((((((((((((((((((((((Diabetes Mellitus, Noninsulin-Dependent[Title/Abstract]) OR (Diabetes Mellitus, Ketosis-Resistant[Title/Abstract])) OR (Diabetes Mellitus, Ketosis Resistant[Title/Abstract])) OR (Ketosis-Resistant Diabetes Mellitus[Title/Abstract])) OR (Diabetes Mellitus, Non Insulin Dependent[Title/Abstract])) OR (Diabetes Mellitus, Non-Insulin-Dependent[Title/Abstract])) OR (Non-Insulin-Dependent Diabetes Mellitus[Title/Abstract])) OR (Diabetes Mellitus, Stable[Title/Abstract])) OR (Stable Diabetes Mellitus[Title/Abstract])) OR (Diabetes Mellitus, Type II[Title/Abstract])) OR (NIDDM[Title/Abstract])) OR (Diabetes Mellitus, Noninsulin Dependent[Title/Abstract])) OR (Diabetes Mellitus, Maturity-Onset[Title/Abstract])) OR (Diabetes Mellitus, Maturity Onset[Title/Abstract])) OR (Maturity-Onset Diabetes Mellitus[Title/Abstract])) OR (Maturity Onset Diabetes Mellitus[Title/Abstract])) OR (MODY[Title/Abstract])) OR (Diabetes Mellitus, Slow-Onset[Title/Abstract])) OR (Diabetes Mellitus, Slow Onset[Title/Abstract])) OR (Slow-Onset Diabetes Mellitus[Title/Abstract])) OR (Type 2 Diabetes Mellitus[Title/Abstract])) OR (Noninsulin-Dependent Diabetes Mellitus[Title/Abstract])) OR (Noninsulin Dependent Diabetes Mellitus[Title/Abstract])) OR (Maturity-Onset Diabetes[Title/Abstract])) OR (Diabetes, Maturity-Onset[Title/Abstract])) OR (Maturity Onset Diabetes[Title/Abstract])) OR (Type 2 Diabetes[Title/Abstract])) OR (Diabetes, Type 2[Title/Abstract])) OR (Diabetes Mellitus, Adult-Onset[Title/Abstract])) OR (Adult-Onset Diabetes Mellitus[Title/Abstract])) OR (Diabetes Mellitus, Adult Onset[Title/Abstract]))) AND ((("Insulin Infusion Systems"[Mesh]) OR ((((((((((((((((((Infusion System,

Insulin[Title/Abstract]) OR (Infusion Systems, Insulin[Title/Abstract])) OR (Insulin Infusion System[Title/Abstract])) OR (System, Insulin Infusion[Title/Abstract])) OR (Systems, Insulin Infusion[Title/Abstract])) OR (Implantable Programmable Insulin Pump[Title/Abstract])) OR (Programmable Implantable Insulin Pump[Title/Abstract])) OR (Pump, Programmable Implantable Insulin[Title/Abstract])) OR (Insulin Pump, Programmable Implantable[Title/Abstract])) OR (Pancreas, Artificial Endocrine[Title/Abstract])) OR (Artificial Endocrine Pancreas[Title/Abstract])) OR (Endocrine Pancreas, Artificial[Title/Abstract])) OR (beta Cell, Artificial[Title/Abstract])) OR (Artificial beta Cell[Title/Abstract])) OR (Artificial beta Cells[Title/Abstract])) OR (Cell, Artificial beta[Title/Abstract])) OR (Cells, Artificial beta[Title/Abstract])) OR (beta Cells, Artificial[Title/Abstract]))) AND (("Dipeptidyl-Peptidase IV Inhibitors"[Mesh]) OR ((((((((((((((((((((((((Dipeptidyl Peptidase IV Inhibitors[Title/Abstract]) OR (DPP-4 Inhibitor[Title/Abstract])) OR (DPP 4 Inhibitor[Title/Abstract])) OR (Inhibitor, DPP-4[Title/Abstract])) OR (DPP-IV Inhibitor[Title/Abstract])) OR (DPP IV Inhibitor[Title/Abstract])) OR (Inhibitor, DPP-IV[Title/Abstract])) OR (DPP-4 Inhibitors[Title/Abstract])) OR (DPP 4 Inhibitors[Title/Abstract])) OR (DPP-IV Inhibitors[Title/Abstract])) OR (DPP IV Inhibitors[Title/Abstract])) OR (Gliptin[Title/Abstract])) OR (Dipeptidyl Peptidase 4 Inhibitor[Title/Abstract])) OR (Dipeptidyl-Peptidase IV Inhibitor[Title/Abstract])) OR (Dipeptidyl Peptidase IV Inhibitor[Title/Abstract])) OR (Inhibitor, Dipeptidyl-Peptidase IV[Title/Abstract])) OR (Dipeptidyl-Peptidase 4 Inhibitor[Title/Abstract])) OR (Inhibitor, Dipeptidyl-Peptidase 4[Title/Abstract])) OR (Dipeptidyl-Peptidase 4 Inhibitors[Title/Abstract])) OR (Dipeptidyl Peptidase 4 Inhibitors[Title/Abstract])) OR (Gliptins[Title/Abstract])) OR (DPP4 Inhibitor[Title/Abstract])) OR (Inhibitor, DPP4[Title/Abstract])) OR (DPP4 Inhibitors[Title/Abstract]))))) AND (randomized controlled trial [Publication Type] OR randomized [Title/Abstract] OR placebo [Title/Abstract])

Table s1 Basic characteristics of the studies included

Study	Country	Course of disease (Y)	Treatment group		Control group		Intervention time (Day)	Outcomes
			N	Interventions	N	Interventions		
Yu 2011[1]	China	≤4	32	B	30	A	28	①②④⑤⑥
Wan 2009[2]	China	0	12/12	B/C	12	A	14	①②③
Ou 2019[3]	China	0	30	C	30	A	20	①②④⑤⑥
Su 2020[4]	China	8	40	C	40	A	15	⑤⑥
Lu 2020[5]	China	/	40/40	D/G	40	A	14	①②⑤
Ma 2021a[6]	China	0	41	F	41	A	14	⑤⑥
Xia 2018[7]	China	0	36	E	36	A	14	①②③⑤
Pan 2006a[8]	China	0	30	B	30	A	28	①②③⑤
Hu 2011[9]	China	0	35	B	32	A	14	①③④
Xue 2012[10]	China	0	30	B	30	A	14	①②③④⑤⑥
Wang 2008[11]	China	≤4	24/26	B/C	23	A	14	①④⑥
Shi 2018[12]	China	0	58	G	58	A	28	①②③④
Li 2015[13]	China	0	89	E	89	A	21	①②③④
Liu 2015[14]	China	5≤Y≤20	24	E	21	A	28	①②③④

Liu 2017[15]	China	0	26	E	26	A	14	①②⑤
Tang 2009a[16]	China	1≤Y≤10	25	C	20	A	14	①②③
He 2013[17]	China	1≤Y≤5	20	C	20	A	14	⑦
Li 2014[18]	China	0	20	C	20	A	14	①②⑤
Xu 2020[19]	China	0	52	C	52	A	14	①②③⑤⑥
Ye 2018[20]	China	2≤Y≤22	52	C	51	A	14	⑤⑥
Jiang 2020[21]	China	0	31	C	31	A	15	①②③⑤⑥
Liang 2012[22]	China	0	25	C	25	A	15	①②④⑤⑥
Chen 2018[23]	China	1≤Y≤13	42	B	42	A	14	①②④⑤⑥
Chen 2008[24]	China	0	29	C	29	A	28	①②③
Dong 2007[25]	China	0	16	B	16	A	28	①②③④⑤
Tang 2009[26]	China	1≤Y≤9	23	D	20	A	14	①②③⑤⑥
Li 2016a[27]	China	0.5≤Y≤21	47	B	47	A	14	①③⑤⑥
Tang 2009b[28]	China	5≤Y≤11	22	B	20	A	14	①②③④⑤⑥
Liao 2018[29]	China	0	50	G	50	A	21	①②③④⑤⑥
Han 2020[30]	China	3.8±1.3	50	F	50	A	14	④⑤⑥
Pan 2006b[31]	China	0	30	B	30	A	7	①②③⑤⑥
Li 2018[32]	China	0	66	B	40	A	14	①②③④⑤⑥

Cao 2019[33]	China	1≤Y≤19	50	C	50	A	21	⑤⑥
He 2014[34]	China	1≤Y≤13	42	B	42	A	14	①②③④
Liang 2013[35]	China	0	21	D	22	A	10	①②⑤
Dou 2009[36]	China	0	17	B	17	A	28	①②③④⑤
Zhi 2013[37]	China	0	40/40	B/C	40	A	14	①②③④
Huang 2017[38]	China	/	27/27	G/D	26	A	14	①②⑤
Li 2016b[39]	China	0	25	G	25	A	14	①②③④
Chen 2019[40]	China	0	40	G	40	A	21	①②③
Guo 2020[41]	China	2≤Y≤9	49	G	49	A	14	①②③⑤⑥
Tan 2020[42]	China	0	30	G	30	A	14	④
Qiao 2020[43]	China	1≤Y≤24	59	G	58	A	28	⑤⑥
Wang 2019[44]	China	/	52	G	49	A	14	①②④⑤⑥
Fang 2019[45]	China	0	25	G	25	A	21	①⑤⑥
Qiu 2020[46]	China	0	38	G	38	A	14	①②
Chen 2017[47]	China	0	40	G	40	A	28	①②⑤⑥
Cheng 2016[48]	China	2≤Y≤9	105	G	105	A	28	①②③⑤⑥
Yan 2021[49]	China	0	37	G	37	A	21	①②⑤⑥
Ma 2016[50]	China	0	34	G	34	A	21	①②⑤⑥

Hu 2016[51]	China	0	30	G	30	A	14	③④
Gao 2018[52]	China	0	35	G	35	A	14	①②⑤⑥
Ma 2021b[53]	China	3≤Y≤8	33	G	33	A	14	①②③④
Wan 2014[54]	China	0	15	G	15	A	14	①②⑤⑥
Heng 2016[55]	China	0	30	G	30	A	14	①②
Guo 2014[56]	China	0	40	G	40	A	14	⑤

Table note: Interventions A:CSII, B:CSII+TZDs, C:CSII+metformin, D:CSII+acarbose, E:CSII+GLP-1 receptor agonist, F:CSII+SGLT-2 inhibitor, G:CSII+DPP-4 inhibitor；Outcome indicators: ①FPG, ②2h-PG, ③HbA1C, ④HOMA-IR, ⑤Insulin dosage, ⑥Time for blood sugar to reach standard.

Table s2 Quality evaluation of the studies included

Study	Randomization generation	Allocation concealment	Blinding	Incomplete data	Selective reporting	Other
Yu 2011[1]	High	Unclear	High	low	low	low
Wan 2009[2]	low	Unclear	low	low	low	low
Ou 2019[3]	low	Unclear	High	low	low	low
Su 2020[4]	low	Unclear	High	low	low	low
Lu 2020[5]	low	Unclear	High	low	low	low
Ma 2021a[6]	low	Unclear	High	low	low	low
Xia 2018[7]	low	Unclear	low	low	low	low
Pan 2006a[8]	High	Unclear	High	low	low	low
Hu 2011[9]	High	Unclear	High	low	low	low
Xue 2012[10]	low	Unclear	low	low	low	low
Wang 2008[11]	low	Unclear	low	low	low	low
Shi 2018[12]	High	Unclear	High	low	low	low
Li 2015[13]	low	Unclear	High	low	low	low
Liu 2015[14]	High	Unclear	High	low	low	low
Liu 2017[15]	High	Unclear	High	low	low	low
Tang 2009a[16]	High	Unclear	High	low	low	low
He 2013[17]	High	Unclear	High	low	low	low
Li 2014[18]	High	Unclear	High	low	low	low
Xu 2020[19]	High	Unclear	High	low	low	low
Ye 2018[20]	High	Unclear	High	low	low	low
Jiang 2020[21]	High	Unclear	High	low	low	low
Liang 2012[22]	High	Unclear	High	low	low	low
Chen 2018[23]	High	Unclear	High	low	low	low
Chen 2008[24]	High	Unclear	High	low	low	low

Dong 2007[25]	High	Unclear	High	low	low	low
Tang 2009[26]	High	Unclear	High	low	low	low
Li 2016a[27]	High	Unclear	High	low	low	low
Tang 2009b[28]	High	Unclear	High	low	low	low
Liao 2018[29]	low	Unclear	High	low	low	low
Han 2020[30]	low	Unclear	High	low	low	low
Pan 2006b[31]	low	Unclear	High	low	low	low
Li 2018[32]	low	Unclear	High	low	low	low
Cao 2019[33]	low	Unclear	High	low	low	low
He 2014[34]	low	Unclear	High	low	low	low
Liang 2013[35]	High	Unclear	High	low	low	low
Dou 2009[36]	low	Unclear	High	low	low	low
Zhi 2013[37]	low	Unclear	High	low	low	low
Huang 2017[38]	High	Unclear	High	low	low	low
Li 2016b[39]	Unclear	Unclear	High	low	low	low
Chen 2019[40]	low	Unclear	High	low	low	low
Guo 2020[41]	Unclear	Unclear	High	low	low	low
Tan 2020[42]	Unclear	Unclear	High	low	low	low
Qiao 2020[43]	Unclear	Unclear	High	low	low	low
Wang 2019[44]	Unclear	Unclear	High	low	low	low
Fang 2019[45]	low	Unclear	High	low	low	low
Qiu 2020[46]	low	Unclear	High	low	low	low
Chen 2017[47]	low	Unclear	High	low	low	low
Cheng 2016[48]	low	Unclear	High	low	low	low
Yan 2021[49]	Unclear	Unclear	High	low	low	low
Ma 2016[50]	low	Unclear	High	low	low	low

Hu 2016[51]	Unclear	Unclear	High	low	low	low
Gao 2018[52]	Unclear	Unclear	High	low	low	low
Ma 2021b[53]	low	Unclear	High	low	low	low
Wan 2014[54]	low	Unclear	High	low	low	low
Heng 2016[55]	low	Unclear	High	low	low	low
Guo 2014[56]	Unclear	Unclear	High	low	low	low

Table s3 Results of network meta-analysis for fasting blood glucose（mmol/L）

**CSII (A)**					
0.60 (0.23, 0.97)[a]	**CSII+TZDs (B)**				
1.25 (0.80, 1.69)[a]	0.65(0.14, 1.16)[a]	**CSII+metformin (C)**			
0.29 (-0.76, 1.36)	-0.31 (-1.42, 0.81)	-0.95 (-2.08, 0.20)	**CSII+acarbose (D)**		
0.68 (-0.06, 1.42)	0.08 (-0.74, 0.90)	-0.56 (-1.43, 0.30)	0.39 (-0.90 1.652)	**CSII+GLP-1RA (E)**	
0.76 (0.37, 1.14)[a]	0.16 (-0.38, 0.70)	-0.48 (-1.08, 0.10)	0.47 (-0.67, 1.58)	0.08 (-0.76, 0.92)	**CSII+DPP-4 inhibitor (G)**

The reported results are displayed with effect size and 95% confidence interval. Mean difference (MD) is applied to continuous results. a $P < 0.05$, with statistical significance.

Table s4 Results of network meta-analysis of 2h-PG（mmol/L）

**CSII (A)**				
0.61 (0.03, 1.19)[a]	**CSII+TZDs (B)**			
2.05 (1.36, 2.76)[a]	1.441(0.61, 2.30)[a]	**CSII+metformin (C)**		
0.81 (-0.73, 2.39)	0.20 (-1.45, 1.88)	-1.24 (-2.92, 0.47)	**CSII+acarbose (D)**	
0.54 (-0.44, 1.53)	-0.08 (-1.20, 1.06)	-1.52 (-2.73, -0.33)[a]	-0.28 (-2.13, 1.54)	**CSII+GLP-1RA (E)**

1.38 (0.85, 1.91)[a]	0.77 (-0.02, 1.56)	-0.67 (-1.56, 0.19)	0.57 (-1.10, 2.19)	0.85 (-0.27, 1.96)	**CSII+DPP-4 inhibitor (G)**

The reported results are displayed with effect size and 95% confidence interval. Mean difference (MD) is applied to continuous results. a P < 0.05, with statistical significance.

Table s5 Results of network meta-analysis of HbA1C（%）

**CSII (A)**					
0.37 (0.07, 0.67)[a]	**CSII+TZDs (B)**				
0.37 (-0.04, 0.76)	0.00 (-0.47, 0.45)	**CSII+metformin (C)**			
0.29 (-0.72, 1.30)	-0.07 (-1.13, 0.97)	-0.07 (-1.16, 1.02)	**CSII+acarbose (D)**		
0.59 (0.08, 1.08)[a]	0.22 (-0.38, 0.80)	0.22 (-0.41, 0.86)	0.30 (-0.85, 1.44)	**CSII+GLP-1RA (E)**	
0.87 (0.55, 1.16)[a]	0.50 (0.06, 0.91)[a]	0.50 (0.00, 0.99)[a]	0.57 (-0.48, 1.63)	0.28 (-0.30, 0.86)	**CSII+DPP-4 inhibitor (G)**

The reported results are displayed with effect size and 95% confidence interval. Mean difference (MD) is applied to continuous results. a P < 0.05, with statistical significance.

Table s6 Results of network meta-analysis of HOMA-IR

**CSII (A)**		
0.33 (0.10, 0.55)[a]	**CSII+TZDs (B)**	
0.59 (0.26, 0.92)[a]	0.27 (-0.09, 0.63)	**CSII+metformin**

		(C)			
0.19 (-0.38, 0.77)	-0.13 (-0.74, 0.50)	-0.40 (-1.06, 0.28)	**CSII+GLP-1RA (E)**		
-0.85 (-1.57, -0.13)[a]	-1.18 (-1.93, -0.42)[a]	-1.45(-2.24, -0.64)[a]	-1.05 (-1.98, -0.13)[a]	**CSII+SGLT-2 inhibitor (F)**	
0.45 (0.17, 0.73)[a]	0.13 (-0.23, 0.49)	-0.14 (-0.57, 0.30)	0.26 (-0.39, 0.89)	**1.303 (0.5326, 2.071)[a]**	**CSII+DPP-4 inhibitor (G)**

The reported results are displayed with effect size and 95% confidence interval. Mean difference (MD) is applied to continuous results. a P < 0.05, with statistical significance.

Table s7 Results of network meta-analysis of insulin dosage (u)

**CSII (A)**						
8.23 (5.84, 10.59)[a]	**CSII+TZDs (B)**					
9.62 (7.10, 12.10)[a]	1.38 (-2.09, 4.83)	**CSII+metformin (C)**				
10.33 (6.60, 14.06)[a]	2.08 (-2.31, 6.51)	0.71 (-3.79, 5.23)	**CSII+acarbose (D)**			
15.17 (9.82, 20.74)[a]	6.94 (1.08, 13.14)[a]	5.56 (-0.28, 11.69)	4.84 (-1.67, 11.56)	**CSII+GLP-1RA (E)**		
11.47 (5.78, 17.11)[a]	3.25 (-2.93, 9.31)	1.85 (-4.38, 8.05)	1.14 (-5.64, 7.91)	-3.71 (-11.71, 4.15)	**CSII+SGLT-2 inhibitor (F)**	
9.06 (7.13, 10.98)[a]	0.84 (-2.20, 3.87)	-0.54 (-3.69, 2.63)	-1.27 (-5.22, 2.69)	-6.11 (-12.01, -0.44)	-2.40 (-8.33, 3.62)	**CSII+DPP-4 inhibitor(G)**

The reported results are displayed with effect size and 95% confidence interval. Mean difference (MD) is applied to continuous results. a P < 0.05, with statistical significance.

Table s8 Results of network meta-analysis of time for blood sugar to reach standard (h)

**CSII (A)**					
1.13 (0.20, 2.05)[a]	**CSII+TZDs (B)**				
2.63 (1.71, 3.55)[a]	1.50 (0.28, 2.74)[a]	**CSII+metformin (C)**			
2.80 (-0.11, 5.75)	1.68 (-1.35, 4.75)	0.18 (-2.87, 3.27)	**CSII+acarbose (D)**		
2.94 (0.99, 4.85)[a]	1.81 (-0.34, 3.94)	0.30 (-1.84, 2.43)	0.13 (-3.39, 3.59)	**CSII+SGLT-2 inhibitor (F)**	
3.36 (2.53, 4.21)[a]	2.24 (1.00, 3.50)[a]	0.73 (-0.51, 1.99)	0.55 (-2.47, 3.57)	0.43 (-1.66, 2.57)	**CSII+DPP-4 inhibitor (G)**

The reported results are displayed with effect size and 95% confidence interval. Mean difference (MD) is applied to continuous results. a $P < 0.05$, with statistical significanc

Table s9 Model fit statistics for all outcomes

Outcome	Model	DIC	Dbar	pD	ratio	I^2	Used in base case analyses
FPG	FE	440.86	386.90	53.96	3.95	75%	No
	RE	187.89	97.39	90.50	0.99	0.4%	Yes
2h-PG	FE	326.50	278.57	47.92	3.24	69%	No
	RE	160.82	84.65	76.16	0.98	0%	Yes
HbA1C	FE	155.39	120.39	35.00	2.00	51%	No
	RE	104.44	55.82	48.62	0.93	0%	Yes
HOMA-IR	FE	297.01	265.00	32.00	4.907	80%	No
	RE	100.31	52.05	48.26	0.96	0%	Yes
Insulin Dosage	FE	402.24	354.20	48.04	4.22	77%	No
	RE	155.34	80.50	74.84	0.96	0%	Yes
Blood sugar	FE	533.05	497.01	36.04	8.02	88%	No
standards time	RE	121.14	61.77	59.37	1.	1%	Yes

DIC, deviance information criterion; FE, fixed effects; RE, random effects.

Table s10 Heterogeneity assessment in network

Outcomes	numbers of trails	number of participants	heterogeneity(I^2)
FPG	46	3395	87.96%
2h-PG	42	3111	86.70%
HbA1C	28	2182	77.26%
HOMA-IR	23	1750	91.16%
Insulin Dosage	38	2903	89.57%
Blood sugar standards time	29	2363	95.75%

Table s11 Assessment of local inconsistencies in different outcome indicators

Outcome indicators	Comparison	Direct WMD (95%CI)	Indirect WMD (95%CI)	Network WMD (95%CI)	P-value
FPG	CSII combined with TZDs vs CSII	-0.52 (-0.92, -0.12)	-1.00 (-2.10, -0.06)	-0.60 (-0.96, -0.22)	0.354
	CSII combined with metformin vs CSII	-1.40 (-1.80, -0.85)	-0.83 (-1.80, 0.16)	-1.20 (-1.70, -0.82)	0.339
	CSII combined with TZDs vs CSII combined with metformin	-0.33 (-1.20, 0.63)	-0.82 (-1.40, -0.18)	-0.65 (-1.20, -0.16)	0.374
2h-PG	CSII combined with TZDs vs CSII	-0.59 (-1.20, 0.02)	-0.77 (-2.70, 1.20)	-0.61 (-1.20, -0.05)	0.870
	CSII combined with metformin vs CSII	-2.10 (-2.80, -1.30)	-2.00 (-3.80, 0.01)	-2.10 (-2.80, -1.30)	0.933
	CSII combined with TZDs vs CSII combined	-1.30 (-3.10, 0.57)	-1.50 (-2.50, -0.58)	-1.50 (-2.30, -0.56)	0.894

	with metformin				
HbA1C	CSII combined with TZDs vs CSII	-0.34 (-0.66, -0.03)	-0.69 (-1.60, 0.31)	-0.37 (-0.68, -0.08)	0.495
	CSII combined with metformin vs CSII	-0.44 (-0.86, 0.03)	-0.12 (-0.98, 0.78)	-0.38 (-0.77, 0.01)	0.524
	CSII combined with TZDs vs CSII combined with metformin	0.22 (-0.62, 1.00)	-0.11 (-0.69, 0.47)	-0.01 (-0.42, 0.45)	0.517
HOMA-IR	CSII combined with TZDs vs CSII	-0.30 (-0.55, -0.04)	-0.55 (-1.20, 0.15)	-0.33 (-0.55, -0.11)	0.485
	CSII combined with metformin vs CSII	-0.67 (-1.10, -0.26)	-0.42 (-1.00, 0.21)	-0.60 (-0.91, -0.24)	0.451
	CSII combined with TZDs vs CSII combined with metformin	-0.11 (-0.64, 0.45)	-0.36 (-0.87, 0.10)	-0.26 (-0.63, 0.09)	0.457
Insulin Dosage	CSII combined with TZDs vs CSII	-11.00 (-15.00, -6.40)	-9.90 (-17.00, -2.70)	-10.00 (-14.00, -6.70)	0.888
	CSII combined with metformin vs CSII	-9.00 (-11.00, -7.10)	-9.60 (-18.00, -1.60)	-9.10 (-11.00, -7.20)	0.875
	CSII combined with TZDs vs CSII combined with metformin	0.79 (-5.90, 8.10)	1.60 (-3.30, 6.70)	1.30 (-2.70, 5.40)	0.852
Blood sugar standards time	CSII combined with TZDs vs CSII	-1.00 (-2.00, -0.04)	-2.10 (-5.00, 0.90)	-1.10 (-2.10, -0.17)	0.511
	CSII combined with metformin vs CSII	-2.70 (-3.70, -1.70)	-1.80 (-4.80, 1.10)	-2.60 (-3.60, -1.70)	0.525

CSII combined with TZDs vs CSII combined with metformin	-0.60 (-3.30, 2.10)	-1.70 (-3.10, -0.39)	-1.50 (-2.80, -0.35)	0.464

The reported results are displayed with effect size and 95% confidence interval (95% CI). Mean difference (MD) is applied to continuous results.

Table s12. PRISMA NMA Checklist of Items to Include When Reporting - A Systematic Review Involving a Network Meta-analysis

Section/Topic	Item #	Checklist Item	Reported on Section, Sub-section, Paragraph #
TITLE			
Title	1	Identify the report as a systematic review incorporating a network meta-analysis (or related form of meta-analysis).	Title
ABSTRACT			
Structured summary	2	Provide a structured summary including, as applicable:<br>Background: main objectives<br><br>Methods: data sources; study eligibility criteria, participants, and interventions; study appraisal; and *synthesis methods, such as network meta-analysis.*<br>Results: number of studies and participants identified; summary estimates with corresponding confidence/credible intervals; *treatment rankings may also be discussed. Authors may choose to summarize pairwise comparisons against a chosen treatment included in their analyses for brevity.*<br>Discussion/Conclusions: limitations; conclusions and implications of findings.<br>Other: primary source of funding; systematic review registration number with registry name.	Abstract
INTRODUCTION			
Rationale	3	Describe the rationale for the review in the context of what is already known, *including mention of why a network meta-analysis has been conducted.*	Introduction; paragraphs 1, 2
Objectives	4	Provide an explicit statement of questions being addressed, with reference to participants, interventions, comparisons, outcomes, and study design (PICOS).	Introduction; paragraph 3
METHODS			
Protocol and registration	5	Indicate whether a review protocol exists and if and where it can be accessed (e.g., Web address); and, if available, provide registration information, including registration number.	Methods; paragraph 1
Eligibility criteria	6	Specify study characteristics (e.g., PICOS, length of follow-up) and report characteristics (e.g., years	Inclusion and exclusion criteria in Methods;

		considered, language, publication status) used as criteria for eligibility, giving rationale. *Clearly describe eligible treatments included in the treatment network, and note whether any have been clustered or merged into the same node (with justification).*	paragraphs 2
Information sources	7	Describe all information sources (e.g., databases with dates of coverage, contact with study authors to identify additional studies) in the search and date last searched.	Retrieval strategy in Methods; paragraph 3
Search	8	Present full electronic search strategy for at least one database, including any limits used, such that it could be repeated.	Supplementary Materials Appendix 1
Study selection	9	State the process for selecting studies (i.e., screening, eligibility, included in systematic review, and, if applicable, included in the meta-analysis).	Paper screening, data extraction, and quality evaluation in Methods; paragraph 4
Data collection process	10	Describe method of data extraction from reports (e.g., piloted forms, independently, in duplicate) and any processes for obtaining and confirming data from investigators.	Paper screening, data extraction, and quality evaluation in Methods; paragraph 4
Data items	11	List and define all variables for which data were sought (e.g., PICOS, funding sources) and any assumptions and simplifications made.	Inclusion and exclusion criteria in Methods; paragraph 2<br>Paper screening, data extraction, and quality evaluation in Methods; paragraph 4
Geometry of the network	S1	Describe methods used to explore the geometry of the treatment network under study and potential biases related to it. This should include how the evidence base has been graphically summarized for presentation, and what characteristics were compiled and used to describe the evidence base to readers.	Statistical analysis in Methods; paragraph 6
Risk of bias within individual studies	12	Describe methods used for assessing risk of bias of individual studies (including specification of whether this was done at the study or outcome level), and how this information is to be used in any data synthesis.	Paper screening, data extraction, and quality evaluation in Methods; paragraph 5
Summary measures	13	State the principal summary measures (e.g., risk ratio, difference in means). *Also describe the use of additional summary measures assessed, such as treatment rankings and surface under the cumulative ranking curve (SUCRA) values, as well as modified approaches used to present summary findings from meta-analyses.*	Statistical analysis in Methods; paragraph 7
Planned methods of analysis	14	Describe the methods of handling data and combining results of studies for each network meta-analysis. This should include, but not be limited to:<br>• *Handling of multi-arm trials;*<br>• *Selection of variance structure;*<br>• *Selection of prior distributions in Bayesian analyses; and*<br>• *Assessment of model fit.*	Statistical analysis in Methods; paragraph 7
Assessment of Inconsistency	S2	Describe the statistical methods used to evaluate the agreement of direct and indirect evidence in the treatment network(s) studied. Describe efforts taken to address its presence when found.	Statistical analysis in Methods; paragraph 7
Risk of bias across studies	15	Specify any assessment of risk of bias that may affect the cumulative evidence (e.g., publication bias, selective reporting within studies).	Statistical analysis in Methods; paragraph 7

Additional analyses	16	Describe methods of additional analyses if done, indicating which were pre-specified. This may include, but not be limited to, the following: • Sensitivity or subgroup analyses; • Meta-regression analyses; • *Alternative formulations of the treatment network; and* • *Use of alternative prior distributions for Bayesian analyses (if applicable).*	Statistical analysis in Methods; paragraph 7
RESULTS†			
Study selection	17	Give numbers of studies screened, assessed for eligibility, and included in the review, with reasons for exclusions at each stage, ideally with a flow diagram.	Literature retrieval in Results; paragraph 1 and Figure 1
Presentation of network structure	S3	Provide a network graph of the included studies to enable visualization of the geometry of the treatment network.	General information of the papers in Results; paragraph 3 and Figure 4
Summary of network geometry	S4	Provide a brief overview of characteristics of the treatment network. This may include commentary on the abundance of trials and randomized patients for the different interventions and pairwise comparisons in the network, gaps of evidence in the treatment network, and potential biases reflected by the network structure.	General information of the papers in Results; paragraph 3 Supplementary Materials Table S1
Study characteristics	18	For each study, present characteristics for which data were extracted (e.g., study size, PICOS, follow-up period) and provide the citations.	Literature retrieval in Results; paragraph 1 Supplementary Materials Table S1
Risk of bias within studies	19	Present data on risk of bias of each study and, if available, any outcome level assessment.	General information of the papers in Results; paragraph 2 and Figures 2, 3 Supplementary Materials Table S2
Results of individual studies	20	For all outcomes considered (benefits or harms), present, for each study: 1) simple summary data for each intervention group, and 2) effect estimates and confidence intervals. *Modified approaches may be needed to deal with information from larger networks.*	Results; Supplementary Materials Table S1
Synthesis of results	21	Present results of each meta-analysis done, including confidence/credible intervals. *In larger networks, authors may focus on comparisons versus a particular comparator (e.g. placebo or standard care), with full findings presented in an appendix. League tables and forest plots may be considered to summarize pairwise comparisons.* If additional summary measures were explored (such as treatment rankings), these should also be presented.	Network meta-analysis results in Results; paragraphs 4, 5, 6, 7, 8 and 9 Figures 5 and 6 Table 1 Supplementary Materials Tables S3, S4, S5, S6, S7 and S8 Figures S1, S2, S3, S4, S5 and S6
Exploration for inconsistency	S5	Describe results from investigations of inconsistency. This may include such information as measures of model fit to compare consistency and inconsistency models, *P* values from statistical tests, or summary of inconsistency estimates from different parts of the treatment network.	Network heterogeneity and inconsistency in Results; paragraph 10 Supplementary Materials Table S11

Risk of bias across studies	22	Present results of any assessment of risk of bias across studies for the evidence base being studied.	General information of the papers in Results; paragraph 2 and Figure 3
Results of additional analyses	23	Give results of additional analyses, if done (e.g., sensitivity or subgroup analyses, meta-regression analyses, *alternative network geometries studied, alternative choice of prior distributions for Bayesian analyses*, and so forth).	
DISCUSSION			
Summary of evidence	24	Summarize the main findings, including the strength of evidence for each main outcome; consider their relevance to key groups (e.g., healthcare providers, users, and policy-makers).	Discussion; paragraphs 1 and 2
Limitations	25	Discuss limitations at study and outcome level (e.g., risk of bias), and at review level (e.g., incomplete retrieval of identified research, reporting bias). *Comment on the validity of the assumptions, such as transitivity and consistency. Comment on any concerns regarding network geometry (e.g., avoidance of certain comparisons).*	Discussion; paragraph 3
Conclusions	26	Provide a general interpretation of the results in the context of other evidence, and implications for future research.	Discussion; paragraph 4
FUNDING			
Funding	27	Describe sources of funding for the systematic review and other support (e.g., supply of data); role of funders for the systematic review. This should also include information regarding whether funding has been received from manufacturers of treatments in the network and/or whether some of the authors are content experts with professional conflicts of interest that could affect use of treatments in the network.	Program for Tianshan Innovative Research Team of Xinjiang Uygur Autonomous Region, China (2020D14020), and the Natural Science Foundation of China (11961071)

Figure s1. The trace map, density map and convergent diagnostic diagram of FPG

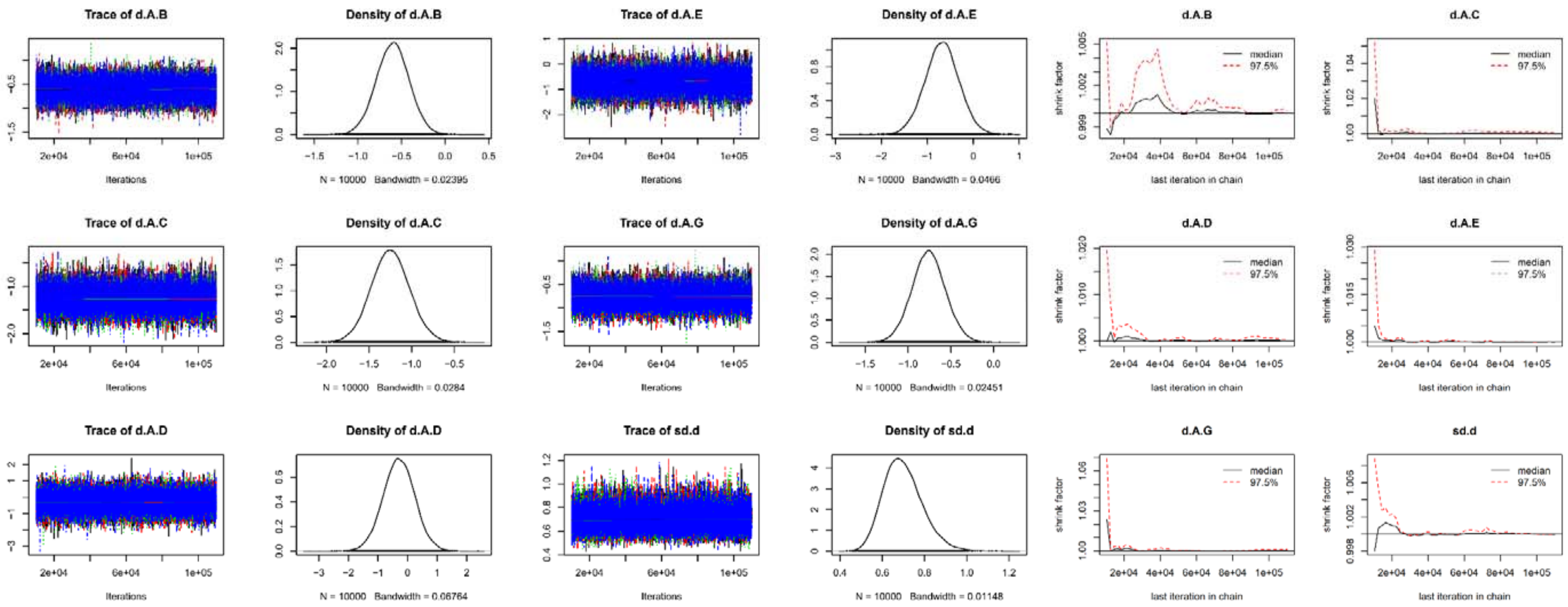

Treatments of the various groups: A represents the group treated with CSII; B represents CSII combined with TZDs; C represents CSII combined with metformin; D represents CSII combined with acarbose; E represents CSII combined with GLP-1 receptor agonist; F represents CSII combined with SGLT-2 inhibitor; G represents CSII combined with DPP-4 inhibitor.

Figure s2. The trace map, density map and convergent diagnostic diagram of 2h-PG

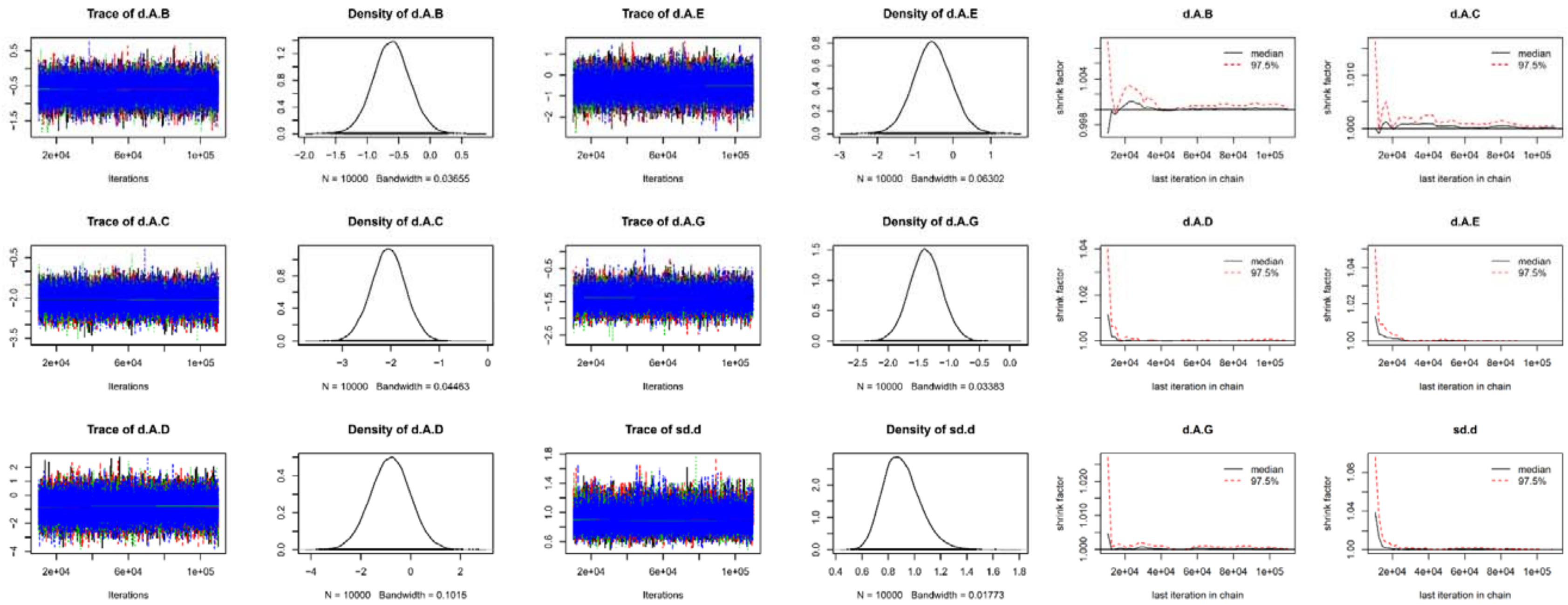

Treatments of the various groups: A represents the group treated with CSII; B represents CSII combined with TZDs; C represents CSII combined with metformin; D represents CSII combined with acarbose; E represents CSII combined with GLP-1 receptor agonist; F represents CSII combined with SGLT-2 inhibitor; G represents CSII combined with DPP-4 inhibitor.

Figure s3. The trace map, density map and convergent diagnostic diagram of HbA1C

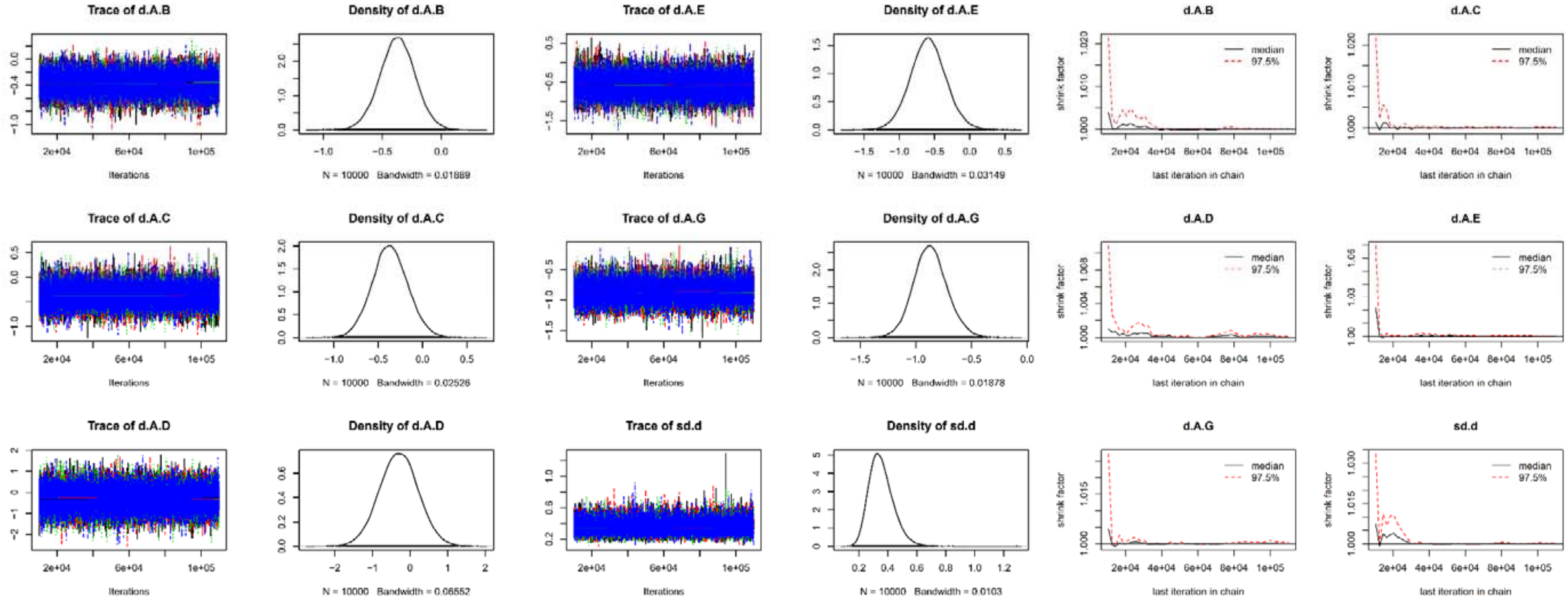

Treatments of the various groups: A represents the group treated with CSII; B represents CSII combined with TZDs; C represents CSII combined with metformin; D represents CSII combined with acarbose; E represents CSII combined with GLP-1 receptor agonist; F represents CSII combined with SGLT-2 inhibitor; G represents CSII combined with DPP-4 inhibitor.

Figure s4. The trace map, density map and convergent diagnostic diagram of HOMA-IR

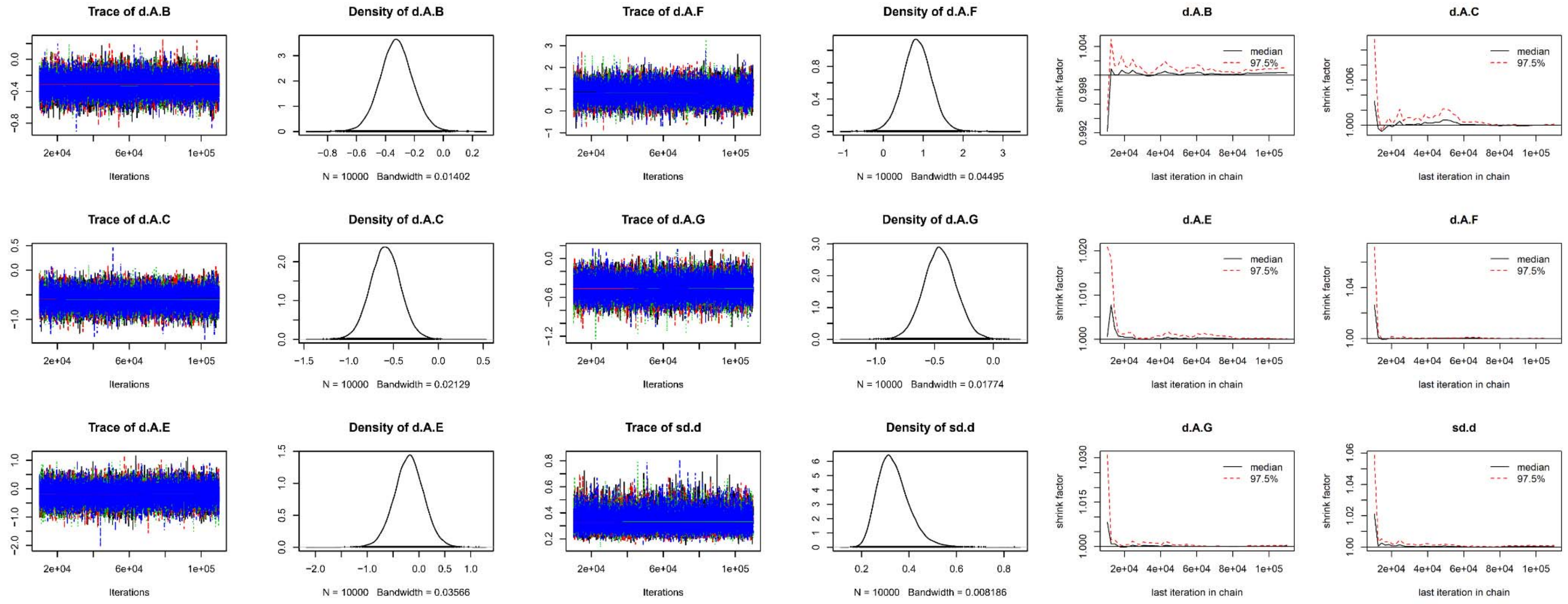

Treatments of the various groups: A represents the group treated with CSII; B represents CSII combined with TZDs; C represents CSII combined with metformin; D represents CSII combined with acarbose; E represents CSII combined with GLP-1 receptor agonist; F represents CSII combined with SGLT-2 inhibitor; G represents CSII combined with DPP-4 inhibitor.

Figure s5. The trace map, density map and convergent diagnostic diagram of insulin dosage

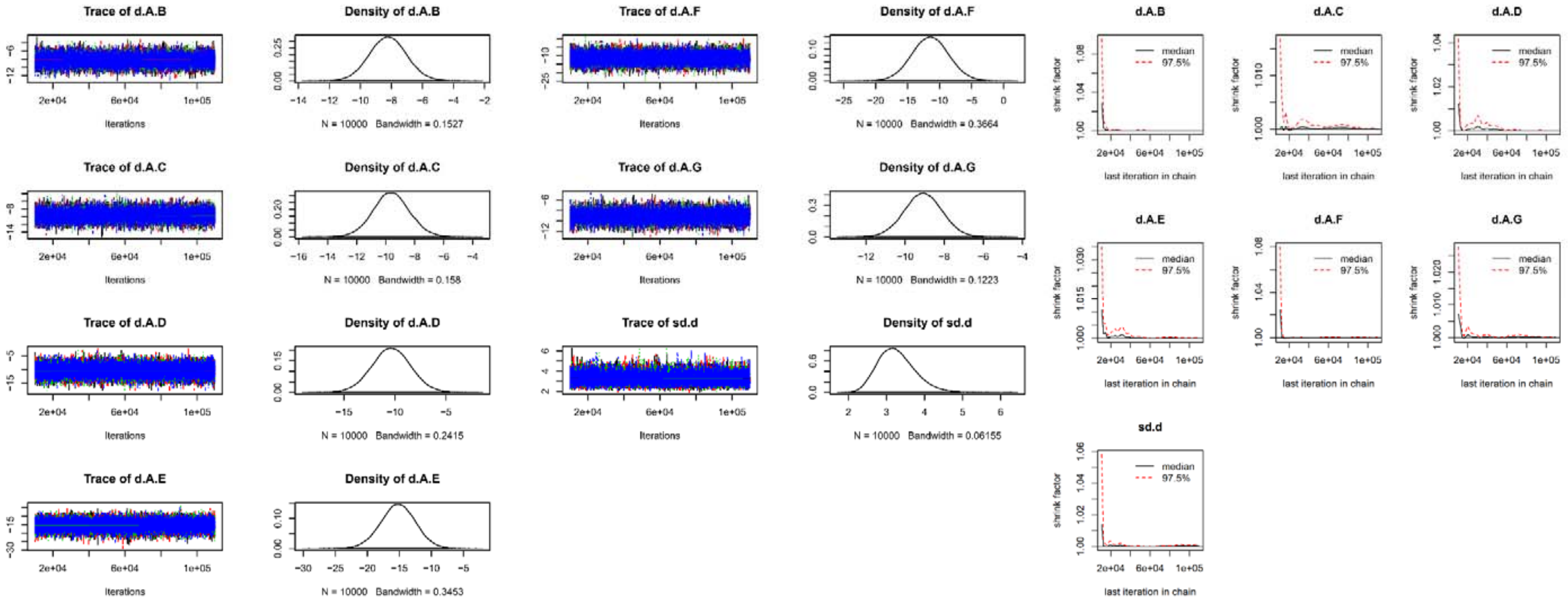

Treatments of the various groups: A represents the group treated with CSII; B represents CSII combined with TZDs; C represents CSII combined with metformin; D represents CSII combined with acarbose; E represents CSII combined with GLP-1 receptor agonist; F represents CSII combined with SGLT-2 inhibitor; G represents CSII combined with DPP-4 inhibitor.

Figure s6. The trace map, density map and convergent diagnostic diagram of blood sugar standards time

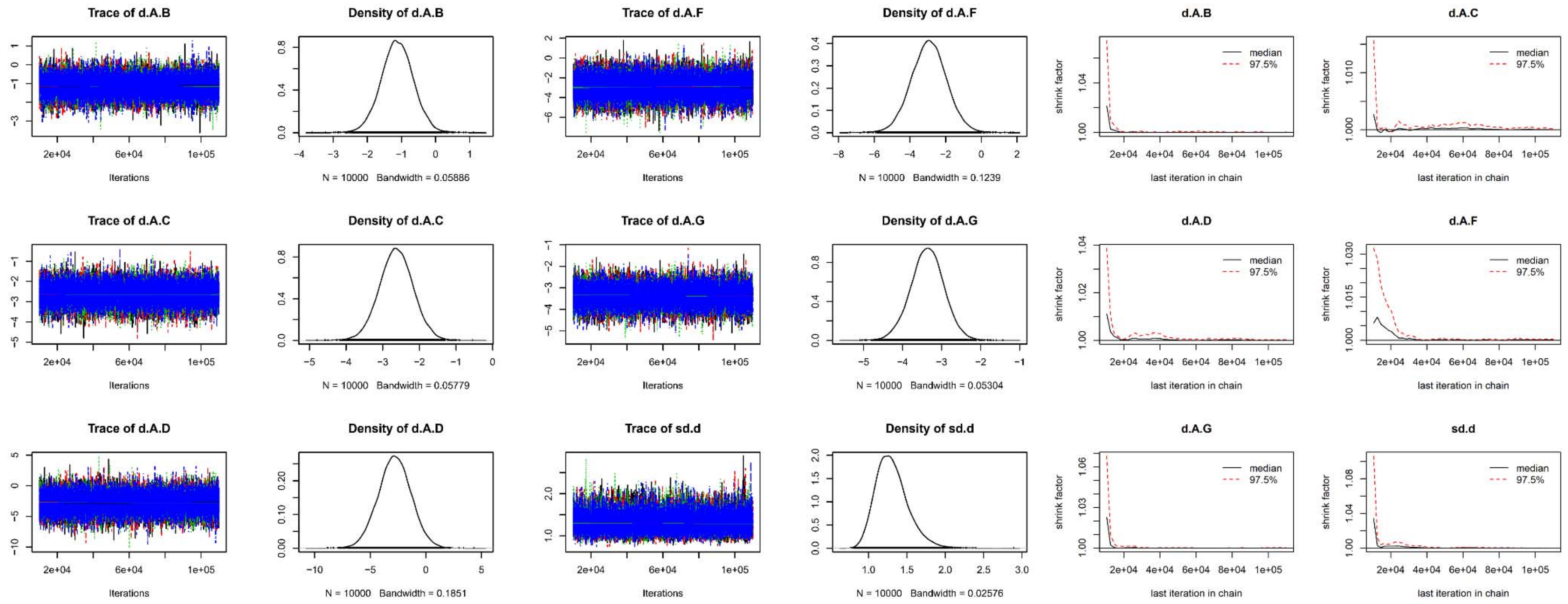

Treatments of the various groups: A represents the group treated with CSII; B represents CSII combined with TZDs; C represents CSII combined with metformin; D represents CSII combined with acarbose; E represents CSII combined with GLP-1 receptor agonist; F represents CSII combined with SGLT-2 inhibitor; G represents CSII combined with DPP-4 inhibitor.

Reference

[1] Yu, H.; Jin, X.; Shen, G. Clinical observation of short-term insulin pump combined with pioglitazone for intensive treatment of primary type 2 diabetes mellitus. *Chinese and foreign health abstract* **2011**;8(23):40-42.

[2] Wan, X. Study of early insulin combination protection against islet cells in patients with newly diagnosed type 2 diabetes. Guangdong: Zhongshan University. **2009**.

[3] Ouyang, R.; Zhang, T.; Zhao, H.; et al. Effect of insulin pump with metformin in the treatment of newly diagnosed type 2 diabetes mellitus. *Science and Education Guide-Electronic edition (early days)* **2019**,3,287.

[4] Su, F. Effect of metformin sustained release tablet in newly diagnosed type 2 diabetes. *Diabetes world* **2020**, 17, 57.

[5] Lu, T. Effect of DPP-4 inhibitors on glycemic control in insulin treatment in patients with type 2 diabetes. *Diabetes world* **2020**, 17, 43.

[6] Ma, J.; Sun, X.; Guo, C. The efficacy of dagliazin combined with insulin pump in patients with type 2 diabetes. *Health nutrition in China* **2021**, 31, 73-74.

[7] Xia, X. Efficacy of liraglutide combined with insulin in first diagnosed overweight and obese T2DM. Henan: Zhengzhou University **2018**.

[8] Pan. H.; Ge, H.; Cai, H. Clinical study on short-term insulin pump combined with rosiglitazone maleate for intensive treatment of primary type 2 diabetes. *Jilin medicine* **2006**, 27, 1381-1382.

[9] Hu, W.; Liu, F.; Yu, W. Effect of pioglitazone on long-term remission of insulin intensive therapy in first diagnosed patients with type 2 diabetes. *Anhuinmedicine* **2011**, 15, 220-222.

[10] Xue, R. Clinical study of rosiglitazone and insulin pump intensification for type 2 diabetes. Jilin: Yanbian University. 2012.

[11] Wang, X. Comparative study of the clinical efficacy of T2DM using CSII and combined with metidin or TZD drugs. Liaonin: Dalian Medical University. 2008.

[12] Shi, X. Ligagliptin and CSII and CSII alone for intensive treatment of primary T2DM Comparative analysis of efficacy. Jilin: Second hospital. 2018.

[13]Li, H. Comparative study of the clinical efficacy of exenatide combination with CSII and CSII alone in patients with naive obese T2DM. Jilin: Yanbian University. 2015.

[14] Liu, X. Clinical effect of liraglutide and insulin pump in non-first diagnosed type 2 diabetes. Shanxi: Yan'an University. 2015.

[15] Liu, J.; Chen, Y.; Lu, Y.; et al. Lyraglutide combined with insulin pump for initial treatment of overweight type 2 diabetes. *The Electronic Journal of Cardiovascular Diseases with Integrated Traditional Chinese and Western Medicine* **2017**, 5, 38.

[16] Tang, Z.; Xu, Z.; Yang, H. Clinical discussion of insulin pump and metformin in intensive treatment for type 2 diabetes mellitus. *Laboratory Medicine and Clinical Medicine* **2009**, 6, 777-778.

[17] He, Z.; Zhang, L. Effect of intensive insulin pump alone and combination of metformin on C peptide. *Clinical Journal of Chinese Medicine* **2013**, 5, 98-99.

[18] Li, H.; Wang, M. Efficacy of Insulin Pump and Metformin in Type 2 Diabetes.*The Journal of Modern Integrated Traditional Chinese and Western Medicine* **2014**, 23, 766-767.

[19] Xu, G.; Zhang, Y.; Zheng, S.; et al. Effect of different oral hypoglycemic drugs on islet function and prognosis in patients with intensive insulin pump treatment for type 2 diabetes. *Western medicine* **2020**, 32, 832-835.

[20] Ye, Y.; Fan, L.; Lu, L. Effect of insulin pump and metformin sustained-release tablets on type 2 diabetes mellitus on insulin resistance. *The new world of diabetes* **2018**, 21, 75-76.

[21] Jiang, Bei.; Xiao, Ping.; Ding, Bo.; et al. Treatment effect of metformin and insulin pump on old new-onset type 2 diabetes mellitus. *The new world of diabetes* **2020**, 23, 75-76,79.

[22] Liang, K.; Lu, Y.; Ou, X.; et al. Effect of metformin sustained release tablet in newly diagnosed type 2 diabetes. *Journal of Practical Medicine* **2012**, 28, 3916-3918.

[23] Chen, Q. Effect of insulin pump intensive therapy and pioglitazone on glycemic control and insulin resistance index changes in type 2 diabetes patients. *Capital Food and Medicine* **2018**, 25, 48.

[24] Chen, H. Clinical observation of insulin pump and metformin in the treatment of primary type 2 diabetes. *Journal of Practical Medical Technology* **2008**, 15, 360-361.

[25] Dong, Z. Study on the treatment of rosiglitazone for type 2 diabetes. *The Practical Diabetes Journal* **2007**, 3, 47.

[26] Tang, Z.; Yang, H.; Xu, Z. Clinical treatment of insulin pump for type 2 diabetes. *The Practical Diabetes Journal* **2009**, 5, 29-30.

[27] Li, D.; Li, Y.; Li, S. Clinical efficacy of insulin pump combined with pioglitazone for intensive short-term treatment in patients with type 2 diabetes. *Journal of the Qiqihar Medical College* **2016**, 37, 2288-2289,2290.

[28] Tang, Z.; Xu, Z.; Yang, H. Clinical treatment of insulin pump and rosiglitazone sodium. *Internal medicine* **2009**, 4, 354-356.

[29] Liao, T.; Li, H.; Deng, B. Clinical efficacy of sitagliptin and insulin pump in the treatment of newly diagnosed type 2 diabetes mellitus. *Journal of Chronic Diseases* **2018**, 9, 1211-1213.

[30] Han, e. Effect of dagliazin on insulin resistance index in patients with short-term intensive insulin pump for type 2 diabetes. *Journal of clinical military medicine* **2020**, 48, 37-438.

[31] Pan, H.; Ge, H.; Cai, H. Clinical study on short-term insulin pump combined with rosiglitazone maleate for intensive treatment of primary type 2 diabetes. *Jilin medicine* **2006**, 27, 1381-1382.

[32] Li, X.; Zhu, L.; Yang, S.; et al. Clinical observation of rosiglitazone combined with insulin pump intensive therapy in improving vascular endothelial function in patients with type 2 diabetes. *North China National Defense Medicine* **2008**, 20, 4-6.

[33] Cao, M.; Wang, T.; Zhu, Y.; et al. Effect of combined insulin pump and metformin sustained-release tablets on type 2 diabetes. *Henan Medical Research* **2019**, 28, 3378-3380.

[34] He, X.; Tang, H. Efficacy of rosiglitazone and insulin pump and its effect on vascular endothelium-dependent relaxation function in type 2 diabetes. *Guangxi medicine* **2014**, 8, 1089-1092.

[35] Liang, H.; Zhang, P.; et al. Effect of acarbose plus insulin pump on blood glucose fluctuations in type 2 diabetes. *Guangxi medicine* **2013**, 35, 453-454.

[36] Dou, N. Clinical observation of insulin pump combined with pioglitazone for primary type 2 diabetes mellitus. *Anhui medicine* **2009**, 13, 665-666.

[37] Huang, Z.; Wan, X.; Liu, J.; Deng, W.; Chen, A.; Liu, L.; Liu, J.; Wei, G.; Li, H.; Fang, D.; Li, Y. Short-term continuous subcutaneous insulin infusion combined with insulin sensitizers rosiglitazone, metformin, or antioxidant α-lipoic acid in patients with newly diagnosed type 2 diabetes mellitus. *Diabetes Technol Ther* **2013**, 15, 859-869.

[38]Huang, H.; Li, .; Cai, X.; et al. Effect of DPP-4 inhibitors on glucose fluctuations in insulin treatment in patients with type 2 diabetes. *Chongqing medicine* **2017**, 46, 2365-2368.

[39]Li, C.; Le, J.; Liu, A.; et al. Effect of early intensive insulin pump therapy on serum Visfatin and GLP-1 in patients with primary type 2 diabetes. *The Journal of Clinical and Experimental Medicine* **2016**, 15, 48-51.

[40]Chen, H. Effect of dipeptidyl peptidase-4 inhibitor in combination with real-time dynamic insulin pump on glycemic control and quality of life in patients with newly diagnosed type 2 diabetes. *Henan Medical Research* **2019**, 28, 3729-3731.

[41]Guo, Ji. Clinical study of dipeptidyl peptidase-4 inhibitor plus insulin pump continuous subcutaneous infusion for insulin asate 30 poorly controlled type 2 diabetes. *The Practical Diabetes Journal* **2020**, 16, 128-129.

[42]Tan, J.; Guo, A. Effect of ligagliptin and insulin pump on blood glucose fluctuations in patients with primary type 2 diabetes. *Traffic medicine* **2020**, 34, 37-38,43.

[43]Qiao, Y. Efficacy observation of two intensive insulin treatment regimen for type 2 diabetes mellitus. *The Continuing Medical Education in China* **2020**, 12, 150-152.

[44]Wang, H.; Chen, P.; Ding, H. Effects of short-term intensive hypoglycemic therapy with insulin pump combined with saxagliptin on secretion function of glandular cells and insulin resistance in patients with type 2 diabetes mellitus. *Drug evaluation study* **2019**, 42, 226-2229.

[45]Fang, M. Effect and adverse effects of sitagliptin in newly diagnosed type 2 diabetes. *Journal of Chronic Diseases* **2019**, 5, 711-713.

[46]Qiu, Q,.; Zhang, G. Efficacy analysis of sitagliptin and insulin pump for short-term intensive therapy in new-onset type 2 diabetes. *Diabetes New World* **2020**, 23, 99-101.

[47]Chen, R.; Chen, Q. Effect of sitagliptin plus insulin pump on blood glucose fluctuations in newly diagnosed type 2 diabetes. *The new world of diabetes* **2017**, 20, 105-106.

[48]Cheng, Y.; Zhang, M.; Wang, L. Efficacy of sitagliptin combined with insulin pump in intensive treatment of T2DM patients with poorly controlled insulin aspartic 30. *Medical clinical research* **2016**, 33, 2009-2011.

[49]Yan, L. Clinical efficacy of sitagliptin and insulin pump in naive patients with type 2 diabetes. *The Practical Diabetes Journal* **2021**, 17, 60-61.

[50]Ma, S.; Zhang, J.; Zheng, Y. Efficacy of sitagliptin and insulin pump in newly diagnosed type 2 diabetes and its effect on blood glucose fluctuations. *Hainan medicine* **2016**,

27, 2183-2184.

[51] Hu, X. Analysis of insulin pump (insulin) and sitagliptin on T2DM. *Modern Drug Application in China* **2016**, 10, 119-120,121.

[52] Gao, Q. Clinical effect of short-term strengthening of insulin pump combined with sitagliptin in newly diagnosed type 2 diabetes. *The World's Latest Medical Information Digest* **2018**, 37, 117-118.

[53] Ma, X. Effect of insulin pump combined with liagliptin on blood glucose and insulin resistance in patients with type 2 diabetes. *Heilongjiang medicine* **2021**, 34, 106-108.

[54] Wan, H. Preliminary study of optimizing continuous subcutaneous insulin injection with DPP-4 enzyme inhibitors for type 2 diabetes. Guangdong: Southern Medical University. 2014.

[55] Wan, H.; Zhao, D.; Shen, J.; Lu, L.; Zhang, T.; Chen, Z. Comparison of the Effects of Continuous Subcutaneous Insulin Infusion and Add-On Therapy with Sitagliptin in Patients with Newly Diagnosed Type 2 Diabetes Mellitus. *J Diabetes Res* **2016**, 2016, 9849328.

[56] Yuan, G.; Jia, J.; Zhang, C.; Yu, S.; Dong, S.; Ye, J.; Zhu, T.; Tang, B.; Qian, W.; Wang, D.; Yang, L.; Zhou, L.; Mao, C. Safety and efficacy of sitagliptin in combination with transient continuous subcutaneous insulin infusion (CSII) therapy in patients with newly diagnosed type 2 diabetes. *Endocr J* **2014**, 61, 513-521.
